# Mesenchymal stem cells deliver synthetic microRNA mimics to glioma cells and glioma stem cells and inhibit their cell migration and self-renewal

**DOI:** 10.18632/oncotarget.868

**Published:** 2013-02-28

**Authors:** Hae Kyung Lee, Susan Finniss, Simona Cazacu, Efrat Bucris, Amotz Ziv-Av, Cunli Xiang, Kevin Bobbitt, Sandra A. Rempel, Laura Hasselbach, Tom Mikkelsen, Shimon Slavin, Chaya Brodie

**Affiliations:** ^1^ Davidson Laboratory of Cell Signaling and Tumorigenesis, Hermelin Brain Tumor Center, Department of Neurosurgery, Henry Ford Hospital, Detroit, MI; ^2^ Everard and Mina Goodman Faculty of Life Sciences, Bar-Ilan University, Ramat-Gan, Israel; ^3^ Department of Public Health Sciences; ^4^ Barbara Jane Levy Laboratory of Molecular Neuro-Oncology; ^5^ Eugene & Marcia Applebaum Laboratory of Invasion & Molecular Therapeutics and; ^6^ The international center for Cell Therapy and Cancer Immunotherapy (CTCI), Tel Aviv, Israel

**Keywords:** miRNA delivery, mesenchymal stem cells, glioma, exosomes

## Abstract

MicroRNAs (miRNAs) have emerged as potential cancer therapeutics; however, their clinical use is hindered by lack of effective delivery mechanisms to tumor sites. Mesenchymal stem cells (MSCs) have been shown to migrate to experimental glioma and to exert anti-tumor effects by delivering cytotoxic compounds. Here, we examined the ability of MSCs derived from bone marrow, adipose tissue, placenta and umbilical cord to deliver synthetic miRNA mimics to glioma cells and glioma stem cells (GSCs). We examined the delivery of miR-124 and miR-145 mimics as glioma cells and GSCs express very low levels of these miRNAs. Using fluorescently labeled miRNA mimics and in situ hybridization, we demonstrated that all the MSCs examined delivered miR-124 and miR-145 mimics to co-cultured glioma cells and GSCs via gap junction–dependent and independent processes. The delivered miR-124 and miR-145 mimics significantly decreased the luciferase activity of their respected reporter target genes, SCP-1 and Sox2, and decreased the migration of glioma cells and the self-renewal of GSCs. Moreover, MSCs delivered Cy3-miR-124 mimic to glioma xenografts when administered intracranially. These results suggest that MSCs can deliver synthetic exogenous miRNA mimics to glioma cells and GSCs and may provide an efficient route of therapeutic miRNA delivery *in vivo*.

## INTRODUCTION

Malignant glioma, including the anaplastic astrocytoma (AA) and glioblastoma (GBM) are the most common and aggressive primary brain tumors [1]. Current treatment options include surgery, radiation therapy, and chemotherapy [2,3]. Unfortunately, prognosis remains extremely poor and the median survival of 12-14 months for patients with GBM has not changed appreciably. Limitations to therapy include the distinctly infiltrative nature of the tumors, which prevents complete resection and contributes to tumor recurrence [4,5], and the high resistance to radio- and chemotherapy of residual tumor cells and glioma stem cells (GSC) [6-8].

One of the alternative approaches to current therapies of GBM with a potential to target infiltrative tumor cells is the use of stem cells, which exhibit homing to tumor and injury sites in the brain [9,10]. Indeed, numerous studies demonstrated tropism of neural stem cells, unmodified or genetically engineered, to infiltrating glioma cells in the brain. However, despite promising results in experimental animal models, there are major limitations in the use of these cells in clinical settings due to difficulties in harvesting and inability to use autologous cells.

Another source of stem cells which exhibits tropism to tumor cells are the adult human mesenchymal stromal stem cells (MSCs). These cells can be obtained from autologous bone marrow and adipose tissues [11,12] or from placenta and umbilical cord that can be used as off-the-shelf cells [13,14]. These cells can be easily expanded in vitro and genetically modified for therapeutic strategies. It was recently reported that MSCs also exhibit homing abilities which enable them to migrate to sites of injury, inflammation and tumors [15]. Specifically, MSCs have been shown to migrate to sites of experimental GBM where they can deliver cytotoxic compounds and exert anti-tumor effects [16,17]. Recent studies suggest that despite sharing similar markers, MSCs that are derived from different sources exhibit differences in their transcriptome, cytokine profile and biological effects [18,19]. Therefore, MSCs from various sources may be differently compatible for cell therapy in specific clinical indications.

MicroRNAs (miRNAs) are small 20–22-nucleotide-long non-coding RNAs that are expressed in the vast majority of eukaryotes, including humans that inhibit gene expression by preventing translation of their target genes [20]. Over the past several years, it has become evident that the post-transcriptional regulation of gene expression by miRNAs has major implications in various areas of cell biology [21] and that deregulation of various miRNAs is associated with a variety of pathological conditions including the initiation and progression of human cancer [22]. Recent studies demonstrated that impairment of the miRNA regulatory network is one of the key mechanisms in GBM pathogenesis [23]. MiRNA deregulation is involved in numerous processes including cell proliferation, apoptosis, cell cycle regulation, invasion, GSC functions, and angiogenesis [24-26]. Indeed, specific miRNAs such as miR-124 and miR-128 have been reported to be downregulated in GBM and GSCs, whereas the expression of other miRNAs including miR-10b, miR-21 and miR-26a is increased in these tumors as compared to normal brains [27]. Therefore, delivery of specific miRNAs or silencing of overexpressed ones appears to be a potential mode of therapy for these tumors. However, despite the high therapeutic potential of miRNAs, their clinical application is limited mainly due to inefficient delivery systems.

In this study, we examined the ability of MSCs enriched from bone marrow, adipose tissue, umbilical cord and placenta to deliver extracellular synthetic miRNAs to glioma cells and GSCs and the effect of these MSC-derived miRNAs on the function of the recipient tumor cells. We found that MSCs from the different sources delivered synthetic miRNAs to both glioma cells and GSCs via gap junction-dependent and -independent processes. Moreover, the delivered miRNA altered gene expression in the recipient glioma cells and impacted their function.

## RESULTS

### MSCs deliver miRNA mimics to glioma cells

**Figure 1:**
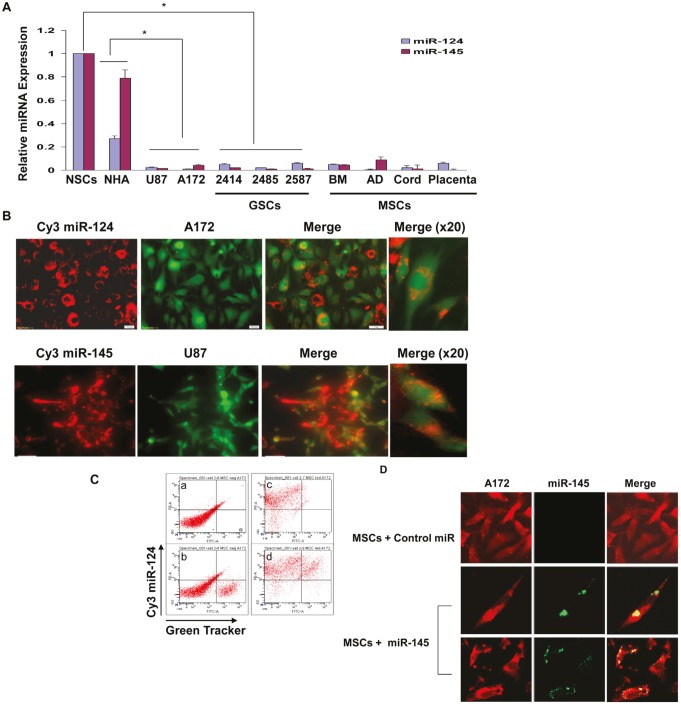
MSCs deliver miRNAs to co-cultured glioma cells The expression of miR-124 and miR-145 was examined in NSCs, normal human astrocytes (NHA), the glioma cells U87 and A172, the GSCs HF2414, HF2485 and HF2587 and in the different MSC preparations using real time PCR (A). BM-MSCs were transfected with Cy3 labeled miR-124 or miR-145 mimics. After 24 h, U87 or A172 cells labeled with Green CellTracker were added to the MSC cultures and the expression of the fluorescent miR-124 in the A172 cells or miR-145 in the U87 cells was analyzed 24 h later using a fluorescence microscope (B). The delivery of Cy3 miR-124 by BM-MSC to the A172 cells was also analyzed using FACS analysis; a - double negative co-cultured cells; b – co-cultured cells with green CellTracker-labeled A172 cells; c – co-cultured cells with MSCs transfected with Cy3-miR-124; d – co-cultured cells with double positive cells (C). BM-MSCs were transfected with a miR-145 mimic and were co-cultured with A172 cells labeled with CellTracker Red for an additional 24 h. In situ hybridization of miR-145 was then performed and the red labeled cells were visualized for the presence of green labeled miR-145. The cells were counted in each of ten random fields (D). The results are the means ± SE of three different experiments (A) or are representative of three different experiments that gave similar results (B-D). *p < 0.001.

In this study we examined the ability of MSCs to deliver exogenous miRNA mimics to the glioma cells U87 and A172 using MSCs from four different sources, bone marrow (BM-MSCs), adipose tissue (AD-MSCs), and MSCs derived from placenta and umbilical cord. In addition, we also employed GSCs derived from GBM specimens. We aimed at delivering miRNAs that are not expressed or expressed in low levels in both the glioma cell lines and the GSCs or in the MSCs, and that their overexpression impacts the function of the tumor cells. Recent studies indicated that miRNA-124 is expressed in low levels in GBM [28]. We therefore first examined the expression of this miRNA in glioma cell lines as compared to human astrocytes (NHA) and in GSCs as compared to neural stem cells (NSCs). Using qRT-PCR, we found that miR-124 was expressed in low levels in the different glioma cell lines and GSCs examined, whereas it was expressed in NSCs and in human astrocytes. Similarly, we found low levels of miR-145 in the GSCs and the glioma cell lines, compared with both NHA and NSCs (Figure 1A). In addition, the different MSCs also express low levels of these miRNAs (Figure 1A).

To examine the ability of MSCs to transfer miRNA mimics to glioma cells we employed miR-124 and miR-145 mimics labeled with Cy3. The MSCs were transfected with the Cy3-miR-124 or Cy3-miR-145 and co-cultured with the specific glioma cell lines that were labeled with a Green CellTracker. After 24-48 h, the cells were viewed under a fluorescence microscope. As presented in Figure 1B, Cy3-miR-124 was detected in the majority of the MSCs (red alone) and in most of the A172 cells labeled with the Green CellTracker. Merged images documented that the transfected MSCs efficiently delivered the miR-124 mimic into the adjacent co-cultured A172 glioma cells albeit to a different degree (Figure 1B). Similar results were obtained with MSCs transfected with Cy3-miR-145 that were co-cultured with the Green CellTracker-labeled U87 cells (Figure 1B). The delivery of the Cy3-miRNA was already observed after 18 h and was maintained in similar levels up to 3 days in co-culture (data not shown).

Using FACS analysis, we further documented that the majority of the glioma cells that were labeled with the Green CellTracker, were also positive for Cy3 miR-124 when co-cultured with BM-MSCs that were transfected with the fluorescent miRNA (Figure 1C).

To further demonstrate the delivery of miRNA mimics by the MSCs, we transfected BM-MSCs with a control miR or a miR-145 mimic and co-cultured these cells with Red CellTracker-labeled A172 cells. Following 24 h we performed in situ hybridization of miR-145 in the glioma cells. As presented in Figure 1D, no detectable expression of miR-145 was observed in A172 cells that were co-cultured with MSCs transfected with a control miR, whereas about 85% of the A172 cells that were co-cultured with MSCs transfected with the miR-145 mimic also expressed this miRNA, further indicating that MSCs transfer exogenous miRNAs to neighboring glioma cells.

We then examined the ability of MSCs from additional sources to deliver miRNAs to glioma cells. The different MSCs expressed similar levels of miR-124 and miR-145 following transfection with the specific miRNA mimics (Supplementary Figure S1). Similar to BM-MSCs, MSCs derived from adipose tissue (AD-MSC), umbilical cord or placenta also efficiently delivered Cy3 miR-124 to glioma cells (Supplementary Figure S2). The transfected MSCs and the fluorescently labeled U87 cells did not exhibit increased levels of cell apoptosis when cultured either alone or together as demonstrated by staining analysis of active caspase 3 or by Western blot analysis of cleaved caspase 3 or PARP (data not shown).

### MSC-derived miR-124 mimic downregulates SCP-1 gene expression in glioma cells

**Figure 2:**
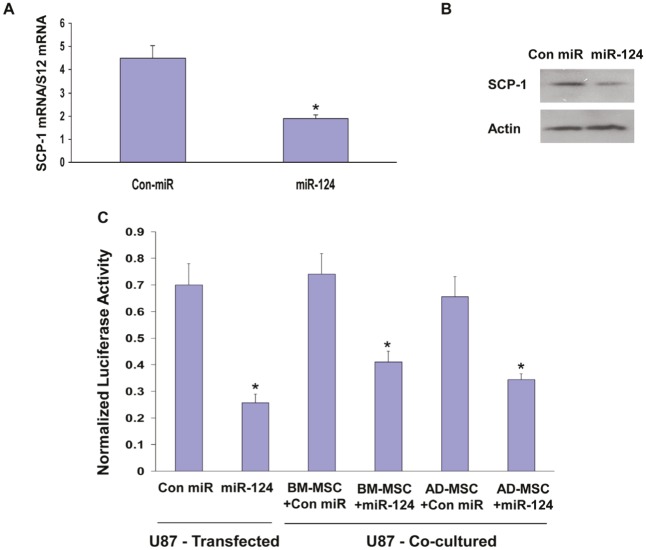
MSC-delivered miR-124 mimic downregulates the expression of SCP-1 in glioma cells U87 cells were transfected with either a control miR (Con-miR) or a miR-124 mimic and the expression of SCP-1 was examined using qRT-PCR (A) or Western blot analysis (B) after 3 days. U87 cells were transfected with a SCP-1 3’-UTR-luciferase plasmid followed by transfection with Con-miR or miR-124 mimic. In parallel, U87 cells expressing this plasmid were co-cultured with BM- MSCs or AD-MSCs that were transfected with either a con-miR or miR-124 mimic (C). The luciferase activity of the cells was determined after 72 h of transfection or co-culture (C). The results are the means ± SE of three different experiments. *p < 0.001.

We then examined if the delivered miR-124 was functional in the recipient glioma cells. miR-124 has been shown to target SCP-1 in various cells [29]. Using qRT-PCR and Western blot analysis, we found that transfection of U87 cells with miR-124 mimic down-regulated the expression of SCP-1 in these cells (Figures 2A, 2B) and A172 cells (data not shown). We also performed a luciferase reporter assay using a construct containing the 3’-UTR of SCP-1 fused to the firefly luciferase reporter gene [29] and found that the transfected miR-124 mimic significantly decreased the luciferase activity of this construct in the U87 cells (Figure 2C). To examine the ability of the MSC-derived miR-124 mimic to target SCP-1 in the recipient glioma cells, we expressed the SCP-1 3’-UTR-luciferase plasmid in the U87 cells and examined the luciferase activity in these cells co-cultured with MSCs transfected with a control miRNA or with the miR-124 mimic. Co-culturing of U87 cells with BM-MSCs expressing a control miR did not affect the luciferase activity of the SCP-1 3’-UTR, whereas co-culturing of U87 cells with BM-MSCs expressing a miR-124 mimic resulted in a significant decrease in activity (Figure 2C). Similar results were observed in U87 cells cultured with AD-MSC expressing a miR-124 mimic (Figure 2C). Therefore, our results that MSC-delivered miR-124 and transfected miR-124 mimic similarly suppress SCP-1 expression demonstrate an efficient functional delivery of miRNA mimics by the co-cultured MSCs.

To exclude the possibility that the inhibitory effect of the miR-124 mimic-transfected MSCs was independent of the specific interaction between the transferred miR-124 and the complementary sites of the SCP-1 3’-UTR luciferase plasmid, we demonstrated that MSCs transfected with a miR-145 mimic, which does not target SCP-1, did not decrease the luciferase activity of this reporter (data not shown).

### Roles of gap junctions and exosomes in the delivery of miR-124 mimic by MSCs

**Figure 3:**
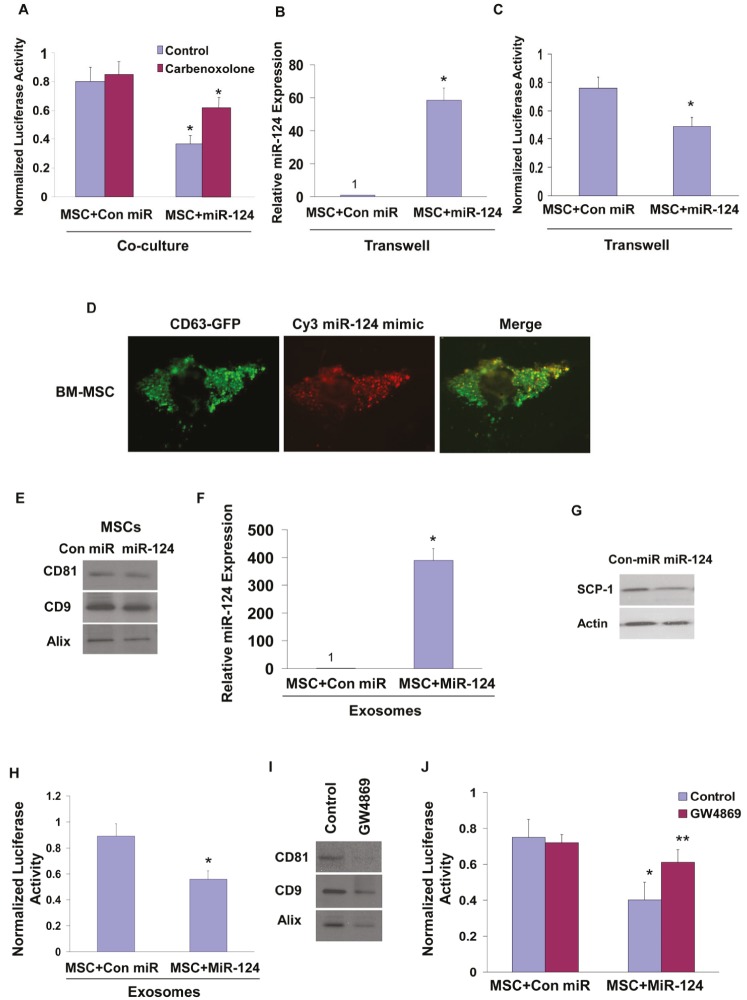
Role of gap junction, exosomes and contact-independent miRNA delivery by MSCs U87 cells transfected with a plasmid expressing SCP-1 3’-UTR-luciferase were co-cultured with BM-MSCs that were transfected with either a con-miR or miR-124 mimic. The cells were treated with carbenoxolone (150 μM) and the luciferase activity of the cells was determined after 48 h of co-culture (A). The contact independent miRNA delivery by MSCs was examined using a transwell chamber with a 0.4 μm filter. MSCs transfected with either a control miR or miR-124 were plated on the upper compartment, whereas U87 cells transfected with SCP-1 3’-UTR-luciferase were seeded on the bottom. Following 48 h, the level of miR-124 was examined in the U87 cells by real time PCR (B) and the luciferase activity was determined as described in Figure 2 (C). BM-MSCs were infected with a lentivirus vector expressing CD63-GFP and transfected with a Cy3 miR-124 mimic. The colocalization of the exosomes and miR-124 was analyzed by a fluorescence microscope and is demonstrated by the yellow dots in the merge image (D). Exosomes were isolated from BM-MSCs using the Exoquick kit or by ultracentrifugation as described in Methods. Both preparations expressed similar levels of CD81, CD9 and Alix (E, Figure S2). The levels of the exosome-delivered miR-124 in U87 cells were determined by real time PCR (F), and the effect of the exosomes on the expression of SCP-1 in these cells was demonstrated by Western blot analysis (G) and by the luciferase activity of U87 cells transfected with the SCP-1 3’-UTR reporter plasmid (H). BM-MSCs (5×10^6^ cells) were treated with GW4869 (5 μM), exosomes were isolated from control and treated cells using the Exoquick kit as described in the methods and the expression of CD81, CD8 and Alix was determined by Western blot analyses (I). BM-MSCs transfected with either control miR or miR-124 were co-cultured with U87 expressing the reporter plasmid SCP-1 3’-UTR. The co-cultures were treated with and without 5 μM GW4869 and the luciferase activity was measured and compared to activity in medium-treated cells (J). The results are the means ± SE of three different experiments (A,B,C,F,H) or are representative of three different experiments (D,E,G,I). *p < 0.001, **p<0.01.

miRNA transfer has been reported to occur via gap junctions in various cell types [30,31]. To examine the role of gap junctions and cell contact in the transfer of synthetic miRNA mimics by MSCs, we first employed the gap junction inhibitor, carbenoxolone [30]. Co-cultures of BM-MSCs transfected with miR-124 mimic and U87 cells transfected with SCP1-3’UTR were treated with carbenoxolone (150 μM) and the luciferase activity of the SCP-1 3’UTR plasmid was examined after 48 h. As presented in Figure 3A, carbenoxolone reduced the inhibition of the luciferase activity induced by the delivered miR-124 mimic by about 50% compared with that of control untreated cells, suggesting that intercellular communication via gap junctions plays at least a partial role in the transfer of functional miRNA mimics between MSCs and glioma cells.

We then examined the role of the contact-independent transfer of miRNA by MSCs. In these studies we employed transwell chambers with 0.4 μm pore-size membranes that do not allow the infiltration of the cells. The miR-124 mimic-transfected MSCs were plated on the upper compartment of the transwell chamber, whereas the labeled U87 cells transfected with the SCP-1 3’ UTR reporter gene were seeded in the bottom one. The ratio of the MSCs to U87 cells was 2:1. Following 48 h, the delivery of miR-124 and the luciferase activity of the reporter gene were examined in the U87 cells. qRT-PCR analysis demonstrated increased delivery of miR-124 to the U87 cells that were cultured with miR-124 transfected MSCs in the transwell chamber (Figure 3B) and a decrease of about 30% in the luciferase activity of the SCP-1 reporter gene (Figure 3C). This decrease was smaller than that observed in the co-cultured cells, however, it clearly demonstrates the presence of contact-independent delivery of miRNA mimics by MSCs to glioma cells.

Intercellular transfer of small RNA has been recently reported to be mediated by exosomes [32]. To determine whether the extracellular miRNA mimics are localized inside exosomes we employed BM-MSCs stably overexpressing the exosomal marker protein CD63 tagged to GFP and transfected them with Cy3-miR-124. The localization of the Cy3-miR-124 and CD63-GFP was then analyzed using a fluorescence microscope. The merge image presented in Figure 3D, demonstrates co-localization of the Cy3 labeled miR-124 and GFP labeled exosomes in the transfected MSCs.

To examine whether the delivery of the miRNA-124 mimic by MSCs is mediated by exosomes, we isolated exosomes from supernatants of MSC expressing miR-124 mimic or a control miRNA. Exosomes were extracted using Exoquick (System Biosciences (Mountain View, CA) or by ultracentrifugation and both preparations expressed similar levels of CD81, CD9 and Alix (Figure 3E, Supplementary Figure S3). Incubation of the U87 cells with MSC-derived exosomes isolated by Exoquick for 24 h resulted in a significant increase in the expression of miR-124 in the U87 cells, whereas exosomes obtained from MSCs expressing a control miRNA did not have any effect on the expression of miR-124 in these cells (Figure 3F, Figure S3). Moreover, we found a decrease in SCP-1 protein expression (Figure 3G) and in the luciferase activity of the SCP-1 3’ UTR reporter plasmid (Figure 3H) in U87 cells treated with exosomes isolated from miR-124-transfected MSCs. Similar effects were obtained with exosomes extracted by ultracentrifugation (Supplementary Figure S3). Based on recent studies [33] and our current results, we used the exosome preparation extracted by Exoquick in our studies.

Exosome secretion has been reported to be triggered by a ceramide-dependent pathway [34,35]. To further demonstrate that the transfer of the miRNA-124 mimic in the MSC-glioma cell co-cultures is mediated by exosomes, we employed the neutral sphingomyelinase (nSMase2) inhibitor GW4869, which blocks ceramide biosynthesis [35]. As presented in Figure 3I, GW4869 significantly decreased the secretion of exosomes as determined by the lower levels of CD81, CD9 and Alix in the exosome preparation of the treated cells. In contrast, the gap junction inhibitor did not have a significant effect on the expression of the exosomal proteins compared to untreated cells (data not shown). Moreover, treatment with GW4869 abolished the decreased luciferase activity of U87 cells transfected with the SCP1-3’-UTR luciferase reporter plasmid and co-cultured with MSCs expressing a miR-124 mimic, whereas it had no effect on the luciferase activity of U87 cells co-cultured with MSCs expressing a control miR (Figure 3J).

Both carbenoxolone and GW4869 did not exert cytotoxic effect as was evident by measurement of LDH (data not shown).

### Delivery of miR-124 mimic by MSCs decreases the migration of glioma cells

**Figure 4:**
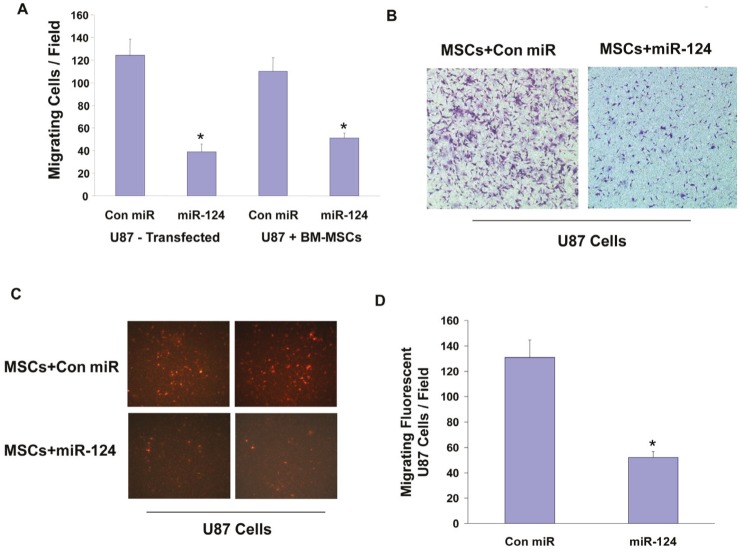
MSC-delivered miR-124 decreases the migration of glioma cells U87 cells were transfected with either control miR or miR-124 mimic and cell migration for 4 h was determined 48 h later using transwell migration (A). U87 cells (A,B) or U87 cells labeled with Red CellTracker (C,D) were cultured with BM-MSCs transfected with either a con-miR or miR-124 mimic. The migration of the co-cultured cells (A,B) and the Red CellTracker labeled U87 cells (C,D) was determined after 48 h using transwell migration assay. The results are representative of three different experiments that gave similar results. *p < 0.001.

We then examined if the miR-124 mimic delivered by MSCs can modulate the function of the co-cultured glioma cells. For these experiments we employed transwell migration assay where cell migration was determined 4 h after plating. Transfection of the U87 cells with a miR-124 mimic decreased the migration of these cells (Figure 4A) as recently described [28]. Similarly, co-cultures of U87 cells with MSCs transfected with a miR-124 mimic had a significant decreased cell migration as determined by a transwell migration assay as compared with co-cultures of U87 cells with MSCs expressing a control miR (Figures 4A, 4B).

As the change in migration in the co-culture experiments could be due to changes in either the MSCs and/or the U87 cells, we further examined the specific migration of the U87 cells by analyzing only the CMPTX (red) CellTracker labeled U87 cells using a fluorescence microscope. As presented in Figures 4C and 4D, the fluorescent U87 cells that were co-cultured with MSCs expressing a miR-124 mimic exhibited a significantly decreased cell migration as compared to cells cultured with MSCs expressing a control miR.

Similar results were obtained with AD-MSCs and with MSCs expressing a non-fluorescent miR-124 (data not shown).

### MSCs deliver miRNA mimics to GSCs and regulate their self-renewal

**Figure 5:**
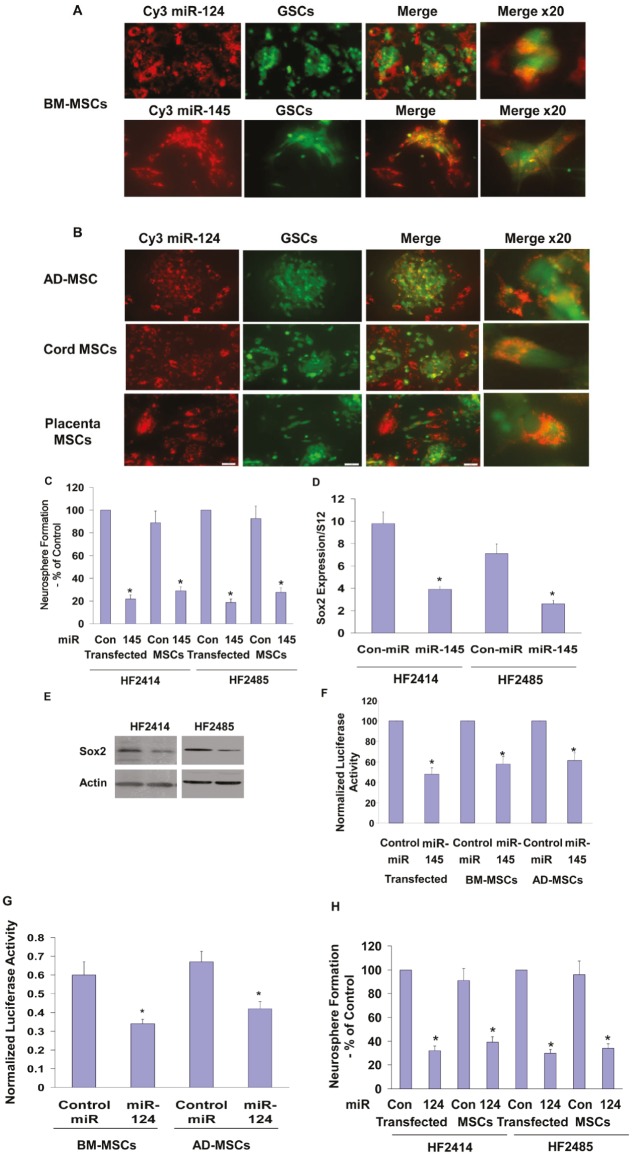
MSCs deliver miRNA mimics to GSCs and decrease their self-renewal BM-MSCs were transfected with Cy3 miR-124 or Cy3 miR-145 mimics (A) and AD-MSCs or MSCs derived from umbilical cord or placenta were transfected with Cy3 labeled miR-124 (B). After 24 h, HF2414 GSCs labeled with Green CellTracker were added to the cultured MSCs for an additional 48 h. The levels of the fluorescent miR-124 and miR-145 (A) and miR-124 (B) were analyzed using a fluorescence microscope. HF2414 or HF2485 GSCs transfected with a control miR or miR-124 mimic (-MSCs) or co-cultured with BM-MSCs transfected with either a control miR, miR-145 (C) or miR-124 (H) mimics were collected after 48 h and analyzed for self renewal for 14 days. HF2414 and HF2485 GSCs were transfected with a miR-145 mimic. After 48 h, the cells were analyzed for the expression of Sox2 mRNA using real time PCR (D) and Sox2 protein using Western blot analysis (E). BM-MSC or AD-MSCs were transfected with a control miR, miR-145 (F) or miR-124 (G) mimics. After 24 h, HF2414 GSCs transfected with either the Sox-2 3’-UTR-luciferase (F) or the SCP-1 3’-UTR-luciferase (G) plasmids were added to the cultured MSCs. The luciferase activity of Sox2-3’UTR (F) or the SCP-1-3’UTR (G) expressed in the GSCs was analyzed after 48 h. The results are representative of three different experiments that gave similar results. *p < 0.001.

GSCs are a rare population of cancer cells that play a role in the migration, resistance to therapy and recurrence of GBM [7,8]. Therefore, targeting these cells is extremely important therapeutically. We found that BM-MSCs delivered both Cy3 miR-124 and Cy3 miR-145 to the GSCs, as evidenced by localization of the Cy3 fluorescent miR in the green labeled HF2414 GSCs (Figure 5A). Similar to the results observed for the miRNA delivery in the glioma cells, we found that MSCs derived from adipose tissue, umbilical cord and placenta also delivered miR-124 to the HF2414 GSCs (Figure 5B) and the HF2485 GSCs (data not shown).

The miR-145 mimic decreased the self-renewal of both HF2485 and HF2414 GSCs (Figure 5C). Likewise, GSCs that were co-cultured with BM-MSCs expressing a miR-145 mimic exhibited a significant decrease in their self-renewal compared to GSCs that were co-cultured with MSCs expressing a control miRNA (Figure 5C).

miR-145 has been recently reported to repress embryonic stem cell pluripotency and the expression of various stemness-related genes including OCT4, Sox2 and KLF4 [36], therefore, we examined the ability of the delivered miR-145 to target Sox2 in the GSCs. We found that transfection of the cells with miR-145 mimic decreased the expression of Sox2 mRNA (Figure 5D) and protein (Figure 5E) in both HF2414 and HF2485 GSCs. In addition, we employed a luciferase reporter plasmid containing the 3’-UTR of Sox2 and found that the miR-145 mimic significantly decreased the luciferase activity of this construct in the HF2414 GSCs (Figure 5F). We further found that the luciferase activity of this construct was significantly decreased in GSCs co-cultured with either BM-MSCs or AD-MSCs transfected with the miR-145 mimic, whereas no significant decrease was observed in the luciferase activity of GSCs co-cultured with MSCs transfected with a control miR (Figure 5F).

We also demonstrated the functional delivery of miR-124 by the BM-MSCs and AD-MSCs to the GSCs by their ability to decrease the luciferase activity of HF2414 GSCs expressing the SCP-1 3’-UTR reporter plasmid when co-cultured together (Figure 5G). In addition, GSCs that were co-cultured with BM-MSCs expressing miR-124 (Figure 5H) or AD-MSCs (data not shown) exhibited a significant decrease in self-renewal as compared to GSCs that were co-cultured with MSCs expressing a control miRNA (Figure 5H).

### MSCs deliver miR-124 to U87-derived xenografts in vivo

**Figure 6:**
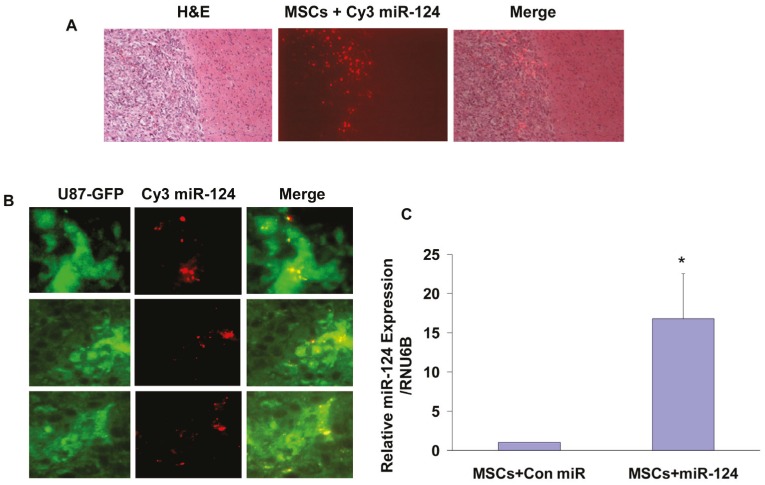
In vivo transfer of Cy3-miR-124 mimic to glioma xenografts by BM-MSCs Glioma xenografts were established from U87-GFP cells. Twenty-two days post tumor cell implantation, BM-MSCs transfected with a Con-miR or with Cy3-mir-124 for 24 h, were injected into the ipsilateral hemisphere (n = 8 for each group), and animals were sacrificed 3 days later. Frozen brain sections were viewed under fluorescence microscopy. H&E staining of frozen sections and fluorescent microscopy demonstrated the presence of MSCs transfected with Cy3-miR-124 in the tumor periphery and within the tumor (A). Merged images (right panels) of GFP-fluorescent tumor cells (left panels) with Cy3-labeled miR-124-transfected MSCs (middle panels) demonstrate Cy3-miR-124 within tumor cells (B). The results are representative of eight different fields that were viewed in each of five different mice (magnification X40). The expression of miR-124 was examined in sorted GFP- positive disaggregated tumor cells derived from 8 mice with U87 xenografts treated with MSCs transfected with a control miR or from 8 mice with U87 xenografts treated with MSCs transfected with Cy3 miR-124 using real-time PCR as described in the methods (C). *p<0.05.

We next examined whether MSCs are capable of delivering miRNA mimics to tumor cells in vivo. For these experiments we established glioma xenografts using U87 cells stably overexpressing pEGFP. Twenty-two days post tumor cell implantation, when the xenografts reached about 60% of their maximal volume, BM-MSCs transfected with a Con-miR or Cy3-miR-124 were injected ipsilaterally and the mice were sacrificed 3 days later. Brain sections were visualized under fluorescent microscopy to analyze the delivery of Cy3-miR-124 by BM-MSCs to the GFP-labeled U87 cells. H&E staining demonstrated the presence of MSCs transfected with Cy3-miR-124 within the xenografts (Figure 6A). After three days, most of the Cy3-labeled MSCs were localized in the peripheral zone of the tumor and some cells within the tumor. Merged images demonstrate the co-localization of Cy3-miR-124 in some of the GFP-labeled glioma cells (Figure 6B), suggesting delivery of the fluorescently labeled miRNA by BM-MSCs to the glioma xenografts.

In addition, we measured the expression of miR-124 in sorted GFP positive tumor cells derived from xenografts treated with MSCs transfected with Con miR or with miR-124. As presented in Figure 6C, the expression of miR-124 was significantly higher in GFP positive tumor cells derived from mice treated with MSCs transfected with miR-124 compared with tumor cells derived from mice treated with MSCs transfecetd with a Con miR.

## DISCUSSION

The present study demonstrates the efficient and functional delivery of exogenous miRNA mimics by MSCs to glioma cells and GSCs via intercellular transfer that alters gene expression and impacts specific glioma cell functions.

GBM, the most malignant and aggressive brain tumors, are characterized by a distinctly invasive nature, which results in residual infiltrative tumor after surgery [5]. In addition, these tumors contain a small rare subpopulation of GSCs that exhibit increased resistance to chemotherapy and radiation therapy and therefore contribute to tumor recurrence [7,8,37].

Novel approaches are urgently being sought for the treatment of GBMs and for the eradication of GSCs including potential clinical applications related to stem cell-based therapy. One of the therapeutic approaches that have recently emerged as potentially promising involves the use of miRNA-based therapies as either miRNA mimics or antagonists depending on the miRNA function and on its status in the specific diseased tissues [38,39]. However, a major drawback for clinical translation of miRNA mimics is the limitations of their targeted delivery to tumor cells [37,39]. In this study we explored the ability of adult MSCs derived from bone marrow, adipose tissues, placenta and umbilical cord to transfer miRNA mimics to co-cultured glioma cells and GSCs as potential vehicles for targeted miRNA-based therapy.

We focused on the delivery of miR-124 and miR-145 since these miRNAs are expressed in low levels in glioma cells and GSCs and therefore can serve as potential candidates for miRNA replacement therapy in GBM. We found that synthetic miR-124 and miR-145 mimics were efficiently delivered to co-cultured glioma cells and GSCs by MSCs derived from bone marrow adipose tissue, cord or placenta as indicated by both the uptake of fluorescent miRNA mimics and by in situ hybridization of the specific miRNA. In addition to the localization of the fluorescent miRNAs in the glioma cells, we also found that the delivered miR-124 mimic was able to modulate gene expression in the recipient cells and to target SCP-1, a known target gene of this miRNA [29].

Intercellular transfer of endogenous miRNAs has been reported in various cellular systems and the importance of this process has been implicated in a variety of both physiological and pathological conditions [40]. Thus, specific miRNAs are transferred between cells of the immune system [32,41] and viral miRNAs secreted by EBV-infected cells can be taken up by uninfected recipient cells [42]. Similarly, MSCs have been reported to secrete and transfer microvesicles containing specific miRNAs which can serve in intercellular communication [43], and to play a role in bone marrow metastases of breast cancer [31]. However, to the best of our knowledge, the use of MSCs for the functional delivery of exogenous synthetic miRNA mimics to cancer cells and cancer stem cells has not been yet described.

miR-124 is downregulated in GBM [28] and its expression is low in both glioma cells and GSCs as compared to astrocytes and NSCs. The overexpression of miR-124 mimic in glioma cells and GSCs decreased their migration and self-renewal, respectively. We also found that miR-145 was expressed in low levels in GSCs and expression of miR-145 mimic in these cells significantly decreased their self-renewal. Similarly, the miR-124 and miR-145 mimics that were delivered by the BM- and AD-MSCs decreased the migration of the U87 cells and the self renewal of the GSCs, respectively, suggesting that the MSCs were able to transfer the exogenous miRNAs in an efficient and functional way.

In addition to the miRNA delivery in vitro, we also found that BM-MSCs that were administered ipsilaterally were able to peripherally penetrate the U87-derived xenografts, deliver the Cy3-miR-124 to nearby glioma cells and downregulate the expression of the miR-124 target gene, CDK6. Studies are underway to characterize the optimal spatial and temporal conditions for miR-delivery by the different MSCs. However, these studies further strengthen the importance of the in vitro results and emphasize the potential role of MSCs as a novel cellular tool for miRNA delivery in vivo.

The mechanisms by which miR-124 regulates cell migration were attributed to its targeting of IQGAP1, laminin γ1 and integrin β1. Similarly, miR-124 was reported to increase the differentiation of GSCs by targeting SCP-1 or CDK6. The inhibitory effects of miR-145 on GSC self renewal may be mediated by the ability of this miRNA to target and decrease the expression of Sox2 and Oct4 [36].

Our results demonstrate that the delivery of the specific miRNA mimics by the MSCs was mediated by exosomes via both gap junction-dependent and contact-independent manner. Transfer by gap junction limits dilution effects and immune responses and it is mediated by connexins that form the gap junction channels [44]. Both MSCs and glioma cells express connexins [44,45] suggesting a possible formation of functional gap junctions between these cells. The MSCs also delivered the miRNA mimics in a contact-independent manner via exosomes, albeit less efficiently. Collectively, these results indicate that MSCs may deliver miRNA mimics efficiently and functionally to both adjacent and remote tumor cells.

Stem cells have been explored as potential vehicles for gene therapy in brain tumors due to their tropism for tumor cells. Indeed, both NSCs and MSCs from both bone marrow and adipose tissue have been shown to migrate successfully to and target glioma cells in preclinical glioma animal models [9-11,12,44,46]. The advantage of autologous BM-MSCs and AD-MSCs as vehicles of miRNA delivery is based on the fact that these cells can be easily obtained and enriched in cultures from each patient and, therefore, allow long-lasting autologous cell therapy with no anticipated complications related to immune rejection. MSCs can be also obtained from additional cellular sources including placenta and Wharton’s jelly of the umbilical cord, thus possibly allowing the use of universal allogeneic cell therapy [14].

We found similar degrees of miRNA transfer by the four types of MSCs examined and similar effects by the BM- and AD-MSCs. AD-MSCs exhibit some advantages over BM-MSCs by being more abundant in the adipose tissue which can be obtained by liposuction and easily expanded and enriched in vitro. However, the use of BM-MSCs has been studied and documented more extensively.

Small RNAs including antisense, siRNA, and miRNA are emerging as promising therapeutic agents against a wide array of diseases [21,23]. Effective delivery of these molecules is crucial to their successful clinical application. MSCs are considered promising vehicles for the delivery of effective and targeted therapies to glioma tumors and glioma infiltrating cells. Our results indicate that in addition to the known ability of MSCs to deliver peptides [47], prodrugs [12,47] and oncolytic viruses [16], they can also deliver synthetic miRNA mimics to glioma cells and GSCs in culture and to glioma xenografts in vivo. These synthetic miRNA mimics can act as physiologically functional molecules to exert gene silencing in mechanisms similar to that of cellular miRNAs. In conclusion, based on our results, the use of MSCs may therefore provide a novel approach for the targeted delivery of anti-cancer miRNAs as a miRNA replacement therapy for GBM and possibly to other types of tumors as well. Studies are currently underway to further characterize the potential use of the different MSCs as chaperones of miRNA delivery in vivo.

## MATERIALS AND METHODS

### Materials

GW4869 was purchased from EMD Biosciences (Billerica, MA) and carbenoxolone was obtained from Sigma (St. Louis, MO). Anti Alix antibody was obtained from Sigma and from Novus Biologicals and anti-CD63, CD81 and CD9 were obtained from System Biosciences (SBI, Mountain View, CA). Normal human astrocytes and neural progenitor cells were obtained from Lonza (Allendale, NJ).

### GSCs and enrichment of CD133+ cells

All human materials were used in accordance with the policies of the Henry Ford Hospital institutional review board (IRB). Generation of the GSCs and enrichment of CD133+ cells and their characterization has been recently described [48-51]. Briefly, GBM specimens were dissociated in 0.05% Trypsin/EDTA for 4 h at room temperature followed by incubation in DMEM/F-12 medium containing 0.7 mg/ml ovomucoid. The tissue was then triturated mechanically with a fire-narrowed Pasteur pipette and filtered through a 40-mm mesh. Cells were density centrifuged in Lympholyte-M and were then maintained in neurosphere medium supplemented with 20 ng/ml EGF and 20 ng/ml FGFb. The enrichment of CD133+ cells was performed according to the MACS CD133 kit manual (Miltenyi Biotech, Auburn, CA). Spheroids were maintained in neurosphere medium and were examined for the expression of CD44, Bmi-1, CD133, Musashi-1, Sox2 and nestin and self-renewal. All the GSCs employed in this study were also examined for the expression of astrocytic, oligodendrocytic and neuronal markers upon plating on poly-D-ornithine in serum-containing medium and for their tumorigenic potential in nude mice or rats as recently described [48,49].

### Mesenchymal stem cell cultures

BM-MSCs, AD-MSCs, placenta and cord-derived MSCs were obtained from ScienCell Research Laboratories (Carlsbad, CA) and were characterized and maintained as previously described [52]. Briefly, the cells were propagated and maintained in Dulbecco’s modified Eagle’s medium (DMEM) supplemented with fetal bovine serum (10%), gentamicin (50 μg/ml), nonessential amino acid (5 mM) and glutamine (5 mM) at 37°C in a 5% CO_2_ humidified atmosphere. Using FACS analysis the cells were found to be positive for CD73, CD90 and CD105 but negative for the hematopoietic markers CD14, CD34, CD80 and CD45. The different cell types were also examined for their ability to differentiate to osteoblasts, chondrocytes and adipocytes. The purity of all the MSC preparations was over 95%.

### Transfection of miRNA mimics

RNA duplexes corresponding to miR-124a (Mission microRNA Mimic hsa-miR-124) and miR-145 (Mission micorRNA Mimic hsa-miR-145) labeled with Cy3 were obtained from Sigma (St. Louis, MO). The control miRNA had no homology for any known human gene sequence and was based on Caenorhabditis elegans sequence. Transfection of the different MSCs with the miRNA duplexes was carried out similarly, with siIMPORTER (Millipore) in 6-well plates according to the manufacturer’s instructions. Briefly, 100 nM of diluted miRNA per well was formulated with siIMPORTER reagent in DMEM serum-free medium. The transfection complex was added directly to the cells and replaced with a fresh medium after 24 h. This method resulted in the transfection of more than 90% of the MSCs as assessed by analysis of the fluorescent miRNAs. In some of the experiments, MSCs were transfected with the miR mimics by electroporation using the Nucleofector device, protocol number U29 (Amaxa Biosystems) to eliminate the presence of residual transfection reagents. Transfection efficiency using nucleofection was over 90% in all the MSCs employed in this study as indicated by fluorescence microscopy. Analyses of the effects of miRNA mimics on recipient cells were performed 2 to 4 days after transfection.

### Co-cultures of MSCs and glioma cells

The green fluorescence CMFDA CellTracker reagent (Molecular Probes, Invitrogen) was used to label glioma cells and GSCs according to the manufacturer’s protocol. Briefly, cells were plated in a 6-well plate and a solution of 5 μM dye was added. After 30 min at 37°C, the dye solution was replaced with fresh medium and the labeled cells were used for co-culture. The Green CellTracker was not transferred from glioma cells to co-cultured MSCs that were transfected with mCherry or labeled with Red CellTracker and no co-localization of green fluorescence was observed in labeled MSCs. The MSCs were transfected with fluorescently labeled microRNA mimics 24 h before the experiment and were washed thoroughly following the transfection to remove residual miRNA and transfection reagent solutions. Green CellTracker-labeled glioma cells or GSCs were added at a ratio of 1:2 to miR-transfected MSCs plated on chamber 8-well slides (ibidi GmbH) and were then analyzed by fluorescence microscopy. In some of the experiments, the transfected MSCs were added to the glioma and GSC cultures.

To examine the contact-independent delivery of the miR-124 mimic we employed transwell chamber with 0.4 μm-pore diameter filters that do not allow cell infiltration. MSCs were transfected with Cy3 labeled miRNAs and the cells were washed thoroughly to remove residual transfection solution 24 h later. The MSCs were plated onto the transwell inserts, whereas the green CMFDA CellTracker-labeled glioma cells were seeded in the lower well of the transwell chambers. After 24-72 h the glioma cells were collected and analyzed for the expression of miR-124 and of SCP-1 expression using qRT-PCR and Western blot analysis.

### Preparation of exosomal fraction

Prior to the collection of culture medium, MSCs transfected with control miR or miR-124 mimic were washed three times with culture medium and were then incubated with OPTIMEM for the experiments of exosome isolation. Exosomes were isolated from the supernatants of MSC cultures following two days of transfection using ExoQuick Exosome precipitation solution (System Biosciences, Mountain View, CA) according to the manufacturer’s instructions. Medium was collected and centrifuged at 3,000 g for 15 min to remove cells and cell debris and was then used for exosome precipitation. Exosomes were pelleted at 1,500 g for 30 min and were then resuspended in 400 μl PBS.

In addition, we also purified exosomes by ultracentrifugation. Supernatant fractions collected from 48 h cell cultures were pelleted by centrifugation at 2000 *g* for 10 min. The supernatant was centrifuged at 20,000*g* for 20 min. Exosomes were then isolated by centrifugation at 100,000 *g* for 70 min at 4^°^C. The exosome pellet was washed in 12 ml of PBS and after additional ultracentrifugation (Sorvall SureSpin 630 rotor) was resuspended in 400 μl PBS.

The Protein content of the enriched exosomal fractions was determined using the Micro BCA assay kit.

### Fluorescence microscopy

Cells were analyzed by fluorescence microscopy (Olympus, Cellsens Dimension) or by a LSM510 Meta confocal microscope equipped with ultraviolet, argon, and helium/neon lasers (Nikon).

### Real-time quantitative PCR analysis

Total RNA was isolated from cultured cells or homogenized tumor sections using QIAzol reagent (Qiagen, CA) according to the manufacturer’s protocol. 0.5 μg of RNA was employed to synthesize cDNA by Thermoscript (Invitrogen) with oligo dT primers. To detect the SCP-1 and SOX2 mRNAs we employed the SYBR green qPCR method using the following primers: SCP-1 - forward CCCAGGACTCAGACAAGATC; reverse CGCTTCAACACGTAGACCTG) and SOX2 forward TGGGTTCGGTGGTCAAGTC; reverse CGCTCTGGTAGTGCTGGGA. CDK6 – forward CTGAATGCTCTTGCTCCTTT; reverse AAAGTTTTGGTGGTCCTTGA

For internal control we employed S12 mRNA levels: forward TGCTGGAGGTGTAATGGACG reverse CAAGCACACAAAGATGGGCT). The expression of miR-124 and miR-145 in the different cells was determined using TaqMan miRNA assays and real-time PCR. All the miRNA assays (hsa-miR-145; 002278, hsa-miR-124a; 000420 and sn-RNU6B; 001973) were obtained from Applied Biosystems (Foster City, CA) and the reactions were run in triplicates. The relative expression of the specific miRNAs was calculated using the comparative Ct method after normalization to snRNU6B. The level of extracellular miRNAs was determined in a fixed volume (500 μl) of culture supernatants and calculated based on their Ct values that were normalized by cel-mir-39: 000200 (Applied Biosystems), which was spiked in each aliquot of the real-time RT–PCR. Quantitative miRNA or mRNA expression data were acquired and analyzed using the ABI Prism 7000 Sequence Detection System (Applied Biosystems). Data were further analyzed by Comparative CT (ΔΔCT) method, and results are expressed in arbitrary units.

### In situ hybridization

In situ hybridization was performed on co-cultures of BM-MSCs transfected with a miR-145 mimic and A172 cells labeled with Red CellTracker. The cells were grown on 18-mm coverslips, fixed with 4% PFA and kept at 4°C in 70% ethanol overnight. The fixed cells were washed with PBS, and then incubated with 0.5% Triton for 10 min. To increase the stability of single-stranded molecules fixed cells were incubated with 40% formamide. Each coverslip was hybridized with 20 ng of the miR 145 probe (has-miR-145 miRCURY LNA^TM^ detection probe, EXIQON). Probes were first diluted in a solution containing SSC, ssDNA/tRNA in 1:1 ratio and 100% formamide. Before hybridization, the solution was boiled at 100°C for 5 min and cooled on ice for another 5 min. The solution was then mixed with a second one containing BSA, SSC and DDW and the 1:1 mixture were applied to the coverslips. The coverslips were incubated in a humidified chamber at 37°C overnight and were washed twice with 40% formamide and incubated in PBS at room temperature for 1 h. Slides were analyzed by confocal microscopy.

### Flow cytometry analysis

MSCs transfected with Cy3-miR-124 for 24 h were co-cultured with A172 cells labeled with Green tracker for an additional 24 h. The cells were collected in PBS without Ca^2+^ and Mg^2+^ and analyzed with a FACSCanto flow cytometer and FACSDiva software (BD Biosciences, Oxford, England). Singlet cells were discerned with a stringent multiparametric gating strategy based on FSC and SSC (pulse width and height). Cells were sorted on a FACSaria flow cytometer (BD Bioscences). The level of fluorescent miR transfer was accessed in the double-positive A172 cells.

Fresh U87-derived xenografts were manually dissociated followed by incubation with a mixture of enzymes including collagenase type III and hyaluronidase. The disaggregated cells were washed and resuspended in phosphate-buffered saline (PBS). GFP-positive cells were sorted and counted using trypan blue staining to exclude dead cells. RNA was extracted as described.

### Luciferase reporter assay

U87 cells or the HF2485 GSCs were co-transfected with the pGL3-SCP1-3’UTR construct (a gift from Prof. Soo-Kyung Lee, Baylor College of Medicine, Houston, Texas) or with the pEZX-MT01-Sox2-3’-UTR construct (GeneCopoeia Rockville, MD) and the pRL-TK Renilla luciferase control vector (Promega (Madison, WI). Transfection was performed by electroporation using the Nucleofector device program A027 and the mouse NSC Nucleofector kit (Amaxa Biosystems, Gaithersburg, MD). The cells were also transfected with miR-124 or a control miRNA using siIMPORTER. After 48 h incubation, Firefly and Renilla luciferase activities were measured using the dual-luciferase assay kit (Promega), according to the manufacturer’s instructions.

### Lentiviral preparation and transduction in MSCs

A Lentivirus-based vector expressing CD63-GFP was purchased from System Biosciences (Mountain View, CA). Lentivirus production and titration were carried out according to the manufacturer’s protocols. Transduction in MSCs was performed in 6-well plates at an MOI of 20.

### Western blot analysis

Cells lysates (30 μg protein) were resolved by SDS-PAGE and transferred to nitrocellulose membranes as previously described [49]. Following incubation with the primary and secondary antibodies the immunoreactive bands were visualized by the ECL Western blotting detection kit (Amersham, Arlington Heights, IL).

### Transwell migration assay

U87 cells labeled with the red-fluorescence CMPTX CellTracker reagent were co-cultured with MSCs that were transfected with miR-124 mimic for one day. After 48 h of co-culture, the cells were analyzed for cell migration using transwell migration assay (pore size, 8 μM) as previously described [50]. Briefly, the co-cultured cells were plated on the upper compartment of the transwell chamber and allowed to migrate to the underside of the top chamber for 3-4 hr. Non-migrated cells on the upper membrane were removed with a cotton swab, and migrated cells attached to the bottom surface of the membrane were fixed, stained and counted. Each experiment was done in triplicates. In addition, the migration of the fluorescently labeled U87 cells was evaluated using a fluorescence microscope.

### Neurosphere formation assay

To determine the ability of CD133+ cells to form secondary neurospheres, we employed GSCs that were labeled with the green fluorescence CMFDA CellTracker reagent and were co-cultured with miR-124 or miR-145 mimic transfected MSCs for 48 h. Spheroids were then collected, disaggregated and plated in 24-well plates at a density of 100 cells/well through limiting dilution, and the number of neurospheres/well was determined 14 days thereafter for 8 different wells as previously described [49,51]. Spheres that contained more than 20 cells were scored. Results are presented as % of maximal neurospheres formed in control untreated cells.

### In vivo miR delivery experiments

Nude female mice (7-9 weeks old, from Taconic) were used in these studies. Animal procedures were approved by the Henry Ford Hospital Animal Care and Use Committee (IACUC). To visualize the xenografts, U87 cells were stably transfected with pEGFP. More than 90% of the cells expressed GFP as accessed by fluorescence microscope following G418 selection. Prior to tumor cell implantation, animals were anesthetized and prepped for sterile surgery. A 1-cm-long incision was made through the scalp, and a 26-gauge needle was used to gently puncture, by twisting, a hole through the skull 2 mm to the right of the bregma and 1 mm anterior to the coronal suture. The needle was lowered 3 mm into the pre-made hole, raised 0.5 mm, and the GFP-labeled U87 cells (3 × 10^5^ cells/5 μl) were injected slowly over 2.5 min. The needle was left in place for 1 min and then slowly raised over 1 min. The animal was removed from the stereotaxic device, the hole was sealed with bone wax, and the scalp sutured. The animals were then monitored for recovery. On day 22 after tumor implantation, when tumor reached about 60% of maximal tumor volume, BM-MSCs that were transfected with a Cy3-labeled miR-124 mimic for 24 h were administered (5 × 10^5^ cells in 5 μl) in the same manner in the ipsilateral hemisphere. After 3 days, when MSCs are expected to migrate to and infiltrate the tumor, standard perfusion with saline was performed and the brains then removed and snap-frozen for sectioning using a cryostat. The delivery of the Cy3-labeled miR-124 by the MSCs was examined in the GFP-labeled glioma cells using a fluorescence microscope.

### Statistical analysis

The results are presented as the mean value ± SE. Data were analyzed using analysis of variance and a Student’s *t* test.

## Supplementary text and figures


