# Supplementary material for: Mesenchymal stem cells deliver synthetic microRNA mimics to glioma cells and glioma stem cells and inhibit their cell migration and self-renewal
Source: Oncotarget. 2013 Feb 28;4(2):346–61. doi: 10.18632/oncotarget.868 (PMC3712579; doi:10.18632/oncotarget.868)
Supplement: Supplementary file 1 [file oncotarget-04-346-s001.pdf]

Supplementary Figure S1

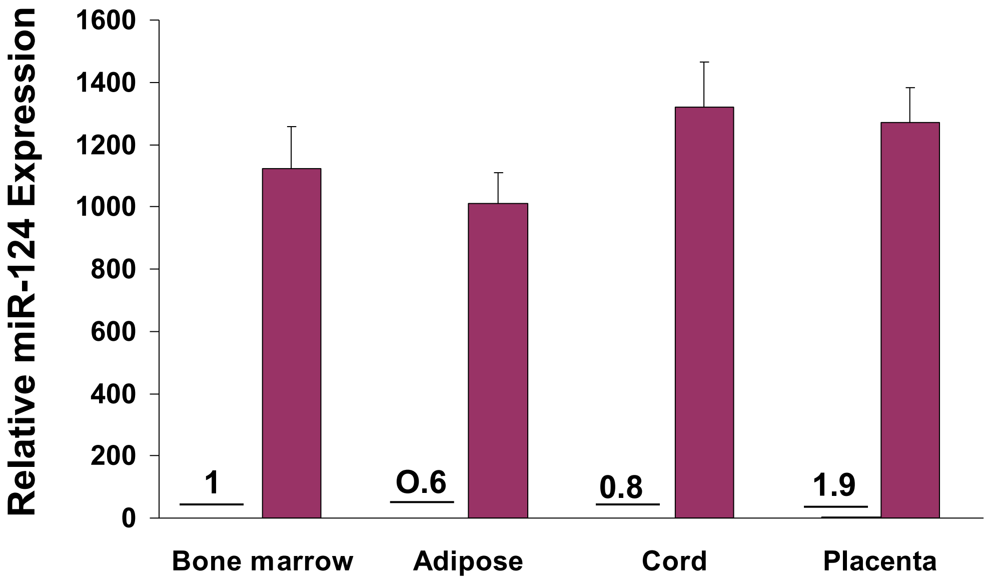

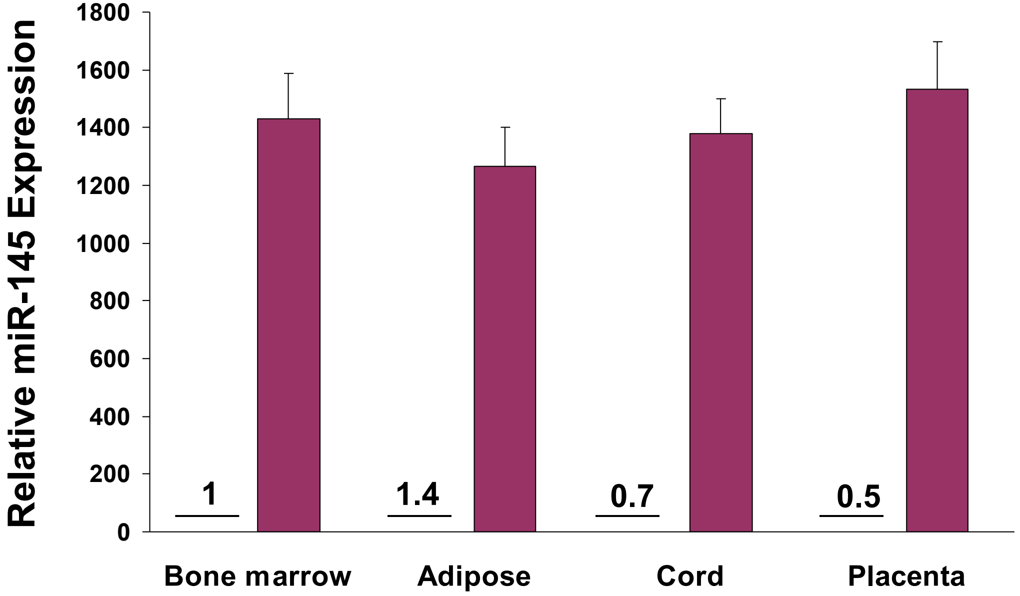

Supplementary Figure S2

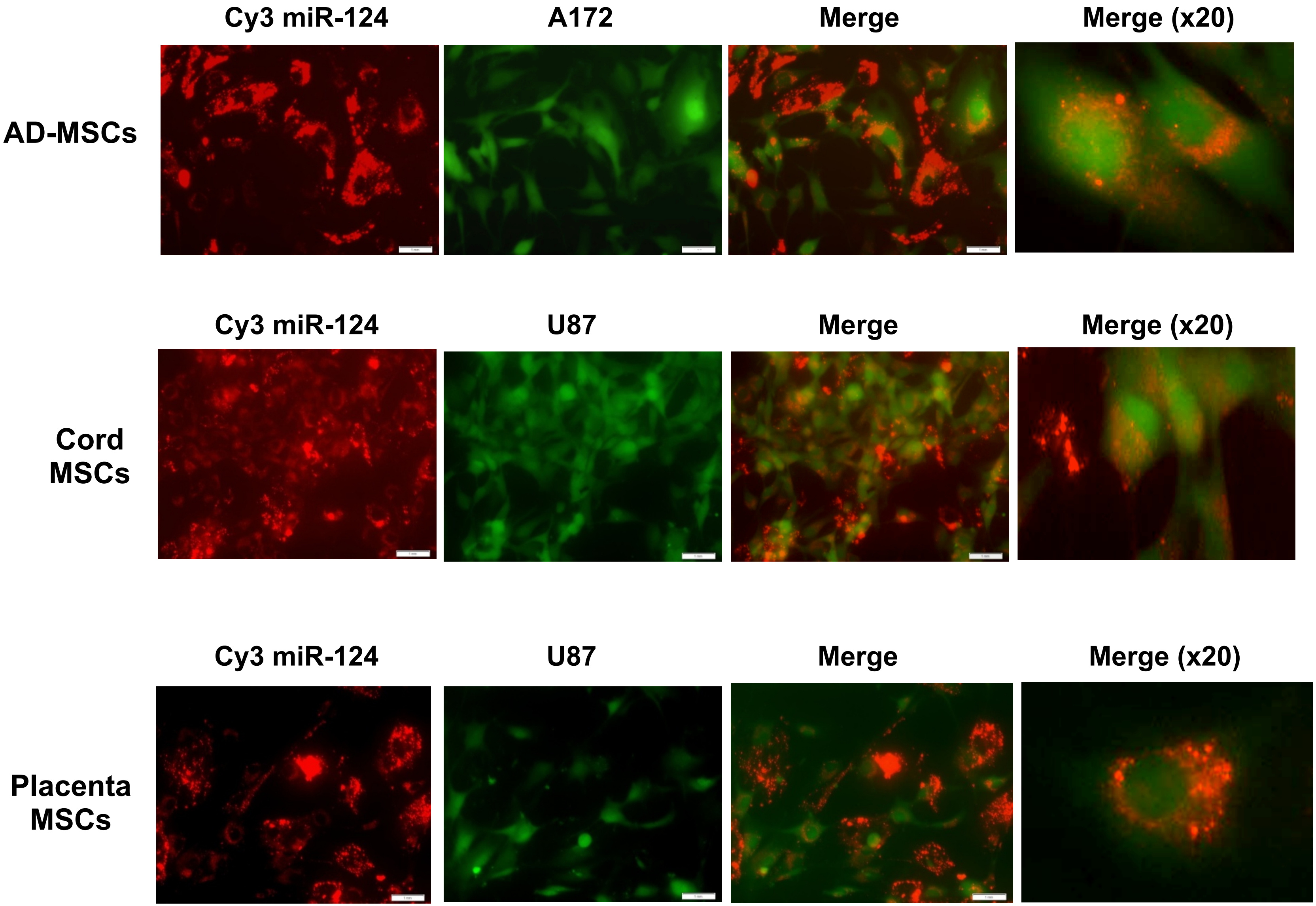

Supplementary Figure S3

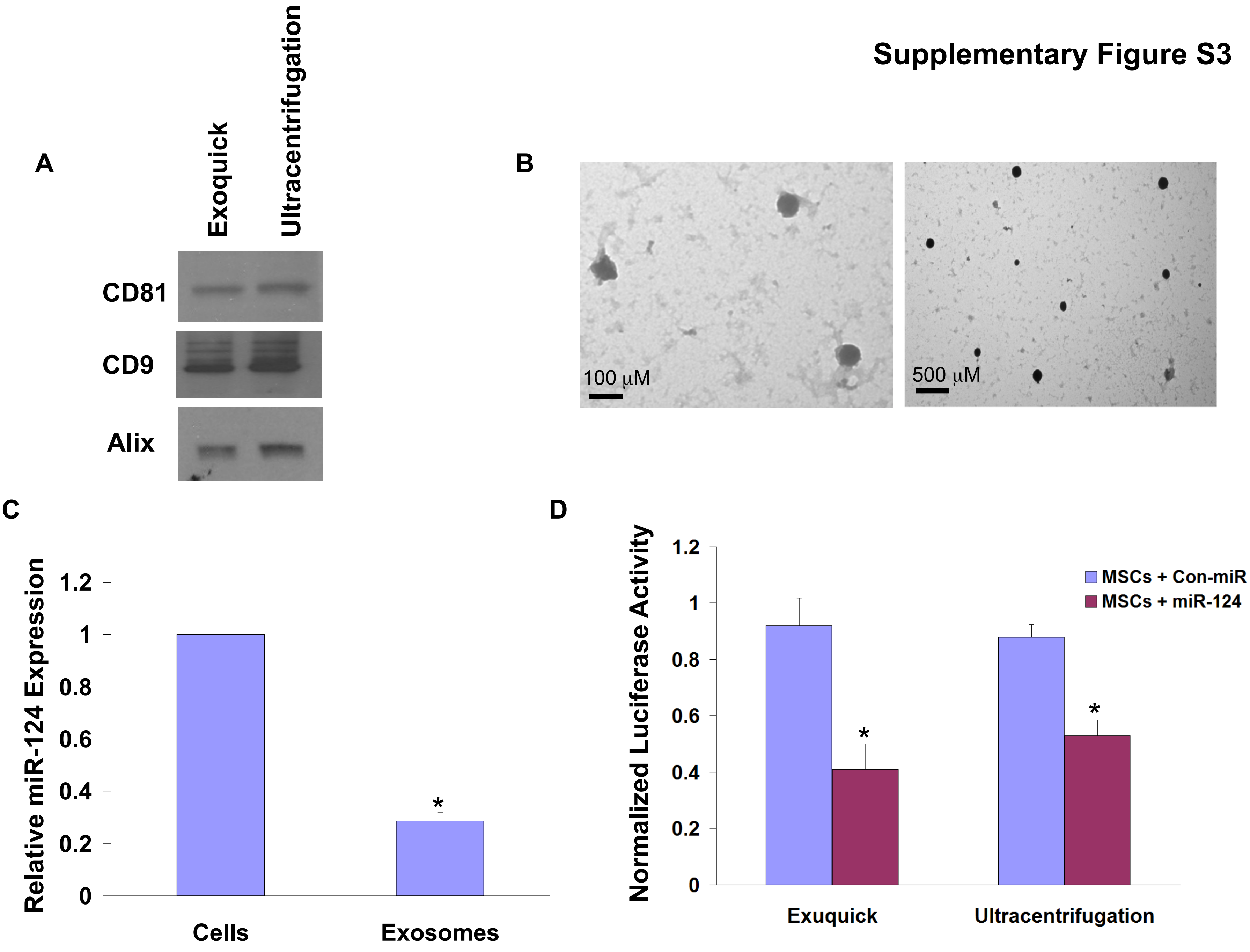

Supplementary figure legends

Figure S1. qRT-PCR analysis of miR-124 and miR-145 levels in transfected MSCs

BM, AD, cord and placenta MSCs were transfected with miR-124 or miR-145 mimics and the levels of the specific miRNAs was determined in the control and transfected cells (red bars) using real time PCR after 48 hr. The results are the means ± SE of triplicate samples of a representative experiment of five different experiments that gave similar results.

Figure S2. MSCs from adipose tissue (AD) cord and placenta deliver miRNAs to co-cultured glioma cells.

MSCs derived from adipose tissue (AD-MSCs), cord or placenta were transfected with Cy3-miR-124 mimic and co-cultured with U87 or A172 cells labeled with Green CellTracker. The delivery of miR-124 to the glioma cells was analyzed using a fluorescence microscope. The results are representative of three different experiments that gave similar results.

Figure S3. Isolation of exosomes from MSCs.

Exosomes were isolated from BM-MSCs using the Exoquick kit or by ultracentrifugation as described in Methods. Both preparations expressed similar levels of CD81, CD9 and Alix (A). TEM images of exosomes isolated by the Exoquick kit demonstrating exosomes in a size range of 40–100 nm (B). Expression levels of miR-124 in BM-MSCs cells transfected with Cy3 miR-124 and exosomes isolated from these cells using real time PCR (C). Effects of exosomes isolated by Exoquick or ultracentrifugation on the luciferase activity of U87 cells transfected with

the SCP-1 3'-UTR reporter plasmid (D). The results are representative of three different experiments that gave similar results (A, B) or are the means ± SE of three different experiments (C). *$p<0.001$
